# Chitin Synthases Are Critical for Reproduction, Molting, and Digestion in the Salmon Louse (*Lepeophtheirus salmonis)*

**DOI:** 10.3390/life11010047

**Published:** 2021-01-13

**Authors:** Hulda María Harðardóttir, Rune Male, Frank Nilsen, Sussie Dalvin

**Affiliations:** 1 Sea Lice Research Centre, Department of Biological Sciences, University of Bergen, P.O. Box 7803, N-5020 Bergen, Norway; rune.male@uib.no (R.M.); frank.nilsen@uib.no (F.N.); 2 Sea Lice Research Centre, Institute of Marine Research, P.O. Box 1870, Nordnes, N-5817 Bergen, Norway; sussie.dalvin@hi.no

**Keywords:** chitin, double-stranded RNA, in situ hybridization, sea lice, aquaculture, ecdysis

## Abstract

Chitin synthase (CHS) is a large transmembrane enzyme that polymerizes Uridine diphosphate *N*-acetylglucosamine into chitin. The genomes of insects often encode two chitin synthases, CHS1 and CHS2. Their functional roles have been investigated in several insects: CHS1 is mainly responsible for synthesizing chitin in the cuticle and CHS2 in the midgut. *Lepeophtheirus salmonis* is an ectoparasitic copepod on salmonid fish, which causes significant economic losses in aquaculture. In the present study, the tissue-specific localization, expression, and functional role of *L. salmonis* chitin synthases, *LsCHS1* and *LsCHS2*, were investigated. The expressions of *LsCHS1* and *LsCHS2* were found in oocytes, ovaries, intestine, and integument. Wheat germ agglutinin (WGA) chitin staining signals were detected in ovaries, oocytes, intestine, cuticle, and intestine in adult female *L. salmonis*. The functional roles of the *Ls*CHSs were investigated using RNA interference (RNAi) to silence the expression of *LsCHS1* and *LsCHS2*. Knockdown of *LsCHS1* in pre-adult I lice resulted in lethal phenotypes with cuticle deformation and deformation of ovaries and oocytes in adult lice. RNAi knockdown of *LsCHS2* in adult female *L. salmonis* affected digestion, damaged the gut microvilli, reduced muscular tissues around the gut, and affected offspring. The results demonstrate that both *Ls*CHS1 and *Ls*CHS2 are important for the survival and reproduction in *L. salmonis*.

## 1. Introduction

The salmon louse*, Lepeophtheirus salmonis,* a parasitic copepod on salmonid fish, is the most commonly found fish louse on salmonids in the Atlantic Ocean [1]. The parasite feeds on host blood, mucus, and skin [2], causing multiple health problems to the host [3,4,5,6]. The salmon louse is an economically important pest in salmon farming and a challenge to the salmon farming industry because of a lack of effective methods to handle the parasite [7,8]. Salmon lice produced on farmed fish spread to wild fish and pose an ecological challenge [9,10,11].

The life cycle of *L. salmonis* consists of eight stages, each separated by a molt [12]. The first three stages (nauplius I and II, and copepodid) are planktonic. The copepodid is the infective stage and becomes parasitic after attachment to a host. The last five life stages (chalimus I and II, pre-adult I and II, and adult stage) are parasitic. The final and last molt is to the adult stage, after which the female undergoes additional growth in the genital segment and abdomen, defined in six maturition stages (T1–T6), during which reproduction is initiated [13]. The oocytes are produced in the ovaries positioned in the cephalothorax and enter the oviduct as previtellogenic oocytes. Vitellogenesis takes place as oocytes enter the genital segment [14]. Males attach spermatophores to the posterior end of the genital segment of females, and eggs are fertilized externally as they are extruded from the genital segment.

Attempts to control *L. salmonis* with anti-sea louse medicines have resulted in emerging resistances [8]; therefore, new tools are needed to control *L. salmonis* infections. These could be medicines targeting processes in *L. salmonis*, which are absent or different in other relevant species. As molting and the formation of an exoskeleton are unique to invertebrates, they are attractive targets for new treatments.

Chitin, a polysaccharide of *N*-acetylglucosamine, is a structural building block of the exoskeleton of arthropods and is synthesized by the enzyme chitin synthase (CHS). Chitin is also present in the peritrophic matrix, a protective layer covering the microvilli of the gut [15,16,17]. *L. salmonis* has a chitin layer in the foregut and hindgut, but not the midgut. Similar to many hemipteran insects the peritrophic matrix has not been found in the midgut [18,19]. Chitin has also been reported in the ovaries, oocytes, embryonic cuticle, and eggs in insects [20,21].

Benzoylureas, also referred to as chitin synthase inhibitors (CSIs), are used to control pests. In Atlantic salmon farming, CSIs are used to control *L. salmonis* and are administered through feeding or bath treatments. In Norway, diflubenzuron and teflubenzuron are the two benzoylureas used in salmon farming. Because these chemicals can harm non-target species in the marine environment, drug use is limited [22]. Chitin has also been reported in aquatic vertebrates, which may also be affected by CSIs [23,24]. Nevertheless, damage to farmed fish caused by CSI treatments has not been reported to our knowledge. The mode of action of this class of drugs is interesting as resistance has never been reported in *L. salmonis* despite many years of use. The mode of action of benzoylurea is not fully understood. Mutation in the *chs1* gene, which changes isoleucine to methionine, leucine, or phenylalanine, was first reported in several strains of a benzoylurea-resistant moth, *Plutella xylostella*, and later documented in benzoylurea-resistant mosquitoes, *Culex pipiens* [25,26,27]. This mutation is positioned in a conserved sequence located in the transmembrane domain, thought to be the translocation site for chitin polymers across the membrane. Furthermore, using the genome-editing method clustered regularly interspaced short palindromic repeats (CRISPR)/Cas9, the mutations were introduced into *Drosophila melanogaster* and conferred significant resistance towards CSIs [25].

Many insects have two CHS gene variants, named CHS1 and CHS2. CHS1 synthesizes chitin in the integument, while CHS2 synthesizes chitin in the peritrophic matrix [28,29,30]. Gene encoding CHS2 has not been found in the hemipteran insect genome, which are also characterized by the absence of the peritrophic matrix. Instead, they have a perimicrovillar membrane: An extracellular layer with a similar function to the peritrophic matrix [21,31]. In decapods, only one type of CHS is reported [32], while for the copepod *Tigriopus japonicus*, three CHSs have been found: One CHS1 and two types of CHS2 [33]. Like insects, *L. salmonis* have two copies of CHS, which have been classified to *Ls*CHS1 and *Ls*CHS2 based on their protein sequences [34]. The tissue-specific expression and exact functional role of these two *Ls*CHSs are not known. In a recent study, knockdown of *LsCHSs* using RNA interference (RNAi) in *L. salmonis* larvae resulted in aberrant and lethal phenotypes when knocking down *LsCHS1*, while *LsCHS2* knockdown had no measurable effect [35]. RNAi-mediated gene silencing in insects has shown that CHS is required for development, growth, reproduction, and digestion [20,21,36,37]. More understanding of CHS can lead to medicine development more specifically directed at salmon louse enzymes.

The present study aimed to enhance the understanding of the role of CHSs during the development and reproduction in *L. salmonis*. Here the expression of *L. salmonis* CHS1 and CHS2 was analyzed in diverse tissues from adult female lice. Furthermore, their transcriptional location by in situ hybridization was determined in adult female lice. Finally, their functional roles in the parasitic stages of *L. salmonis* was obtained by RNAi-mediated gene silencing approach.

## 2. Materials and Methods

### 2.1. Lepeophtheirus salmonis Production

A laboratory strain (*Ls*Gulen) of salmon louse (*Lepeophtheirus salmonis)* was propagated on Atlantic salmon (*Salmo salar*) [38]. The salmon were hand fed on a commercial diet and kept in seawater in standard conditions: A salinity of 34.5 ppt with a temperature of 10 °C. Salmon lice were collected from infected Atlantic salmon anesthetized with a mixture of benzocaine (60 mg/L) and methomidate (5 mg/L) for 3 min. All experiments were done according to the Norwegian Animal Welfare Legislations and the Animal Ethics Committee of the Norwegian Food Safety Authority (ID8589). The fish are not expected to have any adverse effects from the low level of *L. salmonis* infections.

### 2.2. RNA Interference (RNAi) Experiment

#### 2.2.1. Synthesis of dsRNA

Double-stranded RNA (dsRNA) fragments for *LsCHS1* (NCBI GenBank ID MH350851, Ensembl Metazoa EMLSAG00000002853), *LsCHS2* (MH350853, EMLSAG00000007308), and the negative control cod trypsin (*CPY185*) were produced using the MEGAscripts^®^ RNAi Kit (Ambion, Austin, TX, USA) according to the supplier’s instructions with the primers listed in Table 1. Complementary DNA (cDNA) from pre-adult II (for *Ls*CHS1) or adult females (for *Ls*CHS2) were used as templates for PCR production to synthesize the dsRNA fragments. For the negative control, the PCR product was generated from a plasmid containing the cod trypsin (CPY185) fragment. The control fragment has no significant similarity to transcripts expressed in *L. salmonis* [39]. The final concentration of dsRNA was measured using spectrometry (Nanodrop Technologies Inc., Wilmington, DE, USA). The dsRNA fragments were 174 bp long for *LsCHS1*, 564 bp long for *LsCHS2*, and 800 bp for *CPY185*.

#### 2.2.2. Injection of dsRNA Fragments into Pre-Adult I *Lepeophtheirus salmonis*

For the injections, the dsRNA solutions were diluted to 600 ng/μL, and a drop of bromophenol blue (approximately 20 μL) was added to control the injection success visually [39]. The experiments were performed in pre-adult I lice to follow the development to adults. On the first day of the RNAi experiments, the lice were carefully removed from the host fish using tweezers. The dsRNA (approximately 0.5 μL) was injected into the cephalothorax of each louse using borosilicate glass capillaries and pressure from a mouth tube. After the injection, the lice were incubated in seawater for a few hours to recover and then returned to the host fish.

#### 2.2.3. RNAi Trials

**Figure 1:**
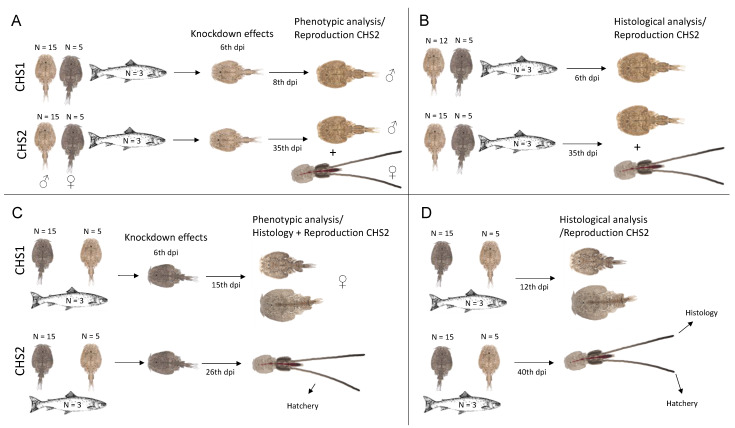
Illustration of the study setup. Trial 1 (**A**) and trial 2 (**B**) are males subjected to RNAi knockdown. Trial 3 (**C**) and trial 4 (**D**) are females subjected to RNAi knockdown. Knockdown of *LsCHS1* (above) and *LsCHS2* (below) for each trial. (**A**,**C**) The first arrows indicate when the knockdown analyses were performed and the second demonstrate the total number of days the lice stayed on the fish before termination. (**A**,**B**) Female with egg strings, reproduction success was studied in females placed with *LsCHS2* knockdown males. (**C**,**D**) Female with egg strings, reproductive success was analyzed in *LsCHS2* knockdown females. *LsCHS1* and *LsCHS2* knockdown lice were terminated as pre-adult II or unmatured adults and mature adults, respectively. Days post-injection = dpi.

Four RNAi experiments were performed, two in males and two in females for both genes (*LsCHS1* and *LsCHS2*) (Figure 1). Trial 1: Males were injected with either ds*LsCHS1* or ds*LsCHS2* to analyze the phenotypic effects on the transcriptional knockdown. Trial 2: Males were injected with either ds*LsCHS1* or ds*LsCHS2* and used for histological analysis. For ds*LsCHS1*, males were harvested in the pre-adult II stage. For ds*LsCHS2*, adult males were harvested. Trial 3: Females were injected with either ds*LsCHS1* or ds*LsCHS2* to analyze the phenotypic effects on the transcriptional knockdown. The knockdown effects on phenotype were analyzed in pre-adult II females, and ds*LsCHS2*-injected adult female lice were collected for histological analysis. Trial 4: Females were injected with ds*LsCHS1* or ds*LsCHS2* and sampled for histological analysis. For *LsCHS1*, pre-molt pre-adult or maturing adult females were harvested. For *LsCHS2*, adult females with the second pair of egg strings were harvested.

#### 2.2.4. Fish Tank Setup

The experiments were performed in standard conditions (Section 2.1) using fish tanks. For trials 1, 3, and 4, fish were placed in small fish tanks (0.07 m^3^), one fish in each tank. Three fish were used for each dsRNA group, and each fish carried ten to 15 injected lice together with five non-injected lice of the opposite sex. For trial 2: Larger fish tanks (0.5 m^3^) were used with five salmon in each. One tank was used for each dsRNA fragment, and each fish carried 12 injected males and five non-injected female lice. Fish tanks were equipped with suitable aquarium nets to collect lice from the water outlet. The lice that fell off the fish and were caught in the net were collected and photographed, and behavioral responses such as swimming and grabbing ability were analyzed. If the lice were normal looking, they were released back into the seawater in the same fish tank. Performing RNAi-mediated knockdown in parasitic stages of *L. salmonis* in fish tanks will lead to small losses of both experimental and control lice due to the natural behavior of the lice. Most of the lice will end up in the net with the water outlet, whilst others are possibly eaten by the fish, or become stuck inside the tank during the experiment. Therefore, fish infected with lice injected with the same dsRNA were placed vertically. Because the water flow goes from top to bottom, any lice that evade the net and fall into the water outlet will end up in a fish tank with lice injected with the same dsRNA.

#### 2.2.5. Sampling and Termination of Trials

The transcriptional levels of target genes (*LsCHS1* or *LsCHS2*) were measured in pre-adult II lice using qPCR (see Section 2.5) to determine the silencing efficiency of dsRNA. For trials 1 and 3: Pre-adult II lice injected with ds*LsCHS2*, ds*LsCHS2*, or ds*CPY* were sampled from fish on the sixth day post-injection. At the end of the experiments, all lice were collected from the host fish and photographed using a Canon EOS 600D camera attached with an adapter (Lmscope) to an Olympus SZX9 dissecting microscope. Subsequently, the lice were fixed in 4% paraformaldehyde or Karnovsky fixative for histological analysis (see Section 2.6 and Section 2.7). The termination points were adjusted for each trial because of the different knockdown effects on the phenotype between *LsCHS1* and *LsCHS2*. For ds*LsCHS1*, trials 1 and 2 were terminated on the eight- and sixth-day post-injection, respectively. Trials 3 and 4 were terminated 13- and 15-days post-injection, respectively. For ds*LsCHS2*, trial 1 and 2 were terminated 35-days post-injection when the dsRNA-injected male lice were adults, and the females had produced the second pair of egg strings. For trial 3, the experiment was terminated 26-days post-injection when the female had produced the first set of egg strings. Trial 4 was terminated 40-days post-injection when the females had produced the second set of egg strings. In trials 1 and 2, both egg strings were removed from untreated adult females and collected into flow-through incubators. In trials 3 and 4, pairs of egg strings were collected from each adult dsRNA-treated female. One egg string was placed into a flow-through incubator, and the other was fixed in 4% paraformaldehyde for histological analysis (see Section 2.7). Hatching ability and the development of the larvae into copepodids were documented. In trials 3 and 4, the total length of the lice was measured. The total length and the morphology of the genital segment were used to determine the life stage of the ds*LsCHS1*-injected lice. For *LsCHS2* knockdown experiments, the total length of the adult females and their egg strings were measured in both the experimental and control groups. The possibility of *LsCHS* knockdown off-target effects was not investigated.

### 2.3. Collection of Tissues and Organs for Tissue-Specific Localization of Transcripts

For tissue-specific gene expression analysis, the organs and tissues were dissected from adult *L. salmonis* females. The body was separated between the cephalothorax and genital segment using a scalpel, and the dorsal integument was carefully stripped from the cephalothorax using tweezers. Then the ovaries were carefully removed from the remaining cephalothorax tissues. Subsequently, the integument covering the genital segment was opened, and the secondary oocytes (hereafter referred to as oocytes) were extracted together with the cement glands. The cement glands were removed from the oocytes using two tweezers: One to separate the oocytes from the cement glands, and the other to remove the cement glands. The intestine was extracted from a new louse. First, the dorsal integument of the cephalothorax was removed, and the genital segment was opened as described above. Intestinal tissue was then removed using tweezers and a scalpel to cut away the tissues attached to the intestine. The tissues around the eye were cut away to get the intestine free from the cephalothorax. The intestine inside the abdomen (the most posterior part of the louse) was collected by cutting away the tissues on both sides of the intestine. The remaining tissues around the intestine were scraped away with tweezers and a scalpel.

### 2.4. RNA Purification and cDNA Synthesis

RNA was extracted from whole lice (pre-adult and adult), egg string pairs, and extracted samples (integument, intestine, and oocytes) from female lice using a Tri Reagent^®^ protocol (Sigma-Aldrich, St. Louis, MO, USA). For homogenization, the lice and tissues (except ovaries, see below) were collected into Eppendorf tubes together with 5 mm stainless steel beads and Trizol (Sigma-Aldrich, St. Louis, MO, USA, 1 mL). The samples were homogenized for 2 min in a TissueLyser II (Qiagen, Hilden, Germany) at a frequency of 30 Hz. The RNA isolation was done according to a Tri Reagent^®^ protocol (Sigma-Aldrich) using 0.2 mL chloroform (Sigma-Aldrich, St. Louis, MO, USA) and the RNA pellets were washed twice in 1 mL 75% ethanol and dissolved in 15 μL of RNAase-free water. The RNA samples were analyzed using Nanodrop 2000 spectrophotometry (Nanodrop Products, Wilmington, Germany) and stored at −80 °C or directly treated with DNase. The DNase treatment was performed according to the manufacturer’s protocol (Turbo DNase free™ kit, Ambion Foster City, CA, USA). The ovaries (three in each sample) were purified using the RNeasy Micro Kit (Qiagen, Hilden, Germany) according to the manufacturer’s recommendations using 1.4 mm zirconium oxide beads to homogenize the samples. The purified RNA was stored at −80 °C before complementary DNA (cDNA) synthesis. For all samples, RNA (200 ng) was reverse transcribed to cDNA using the Affinity Script cDNA Kit for qPCR (Agilent, San Clara, CA, USA) according to the manufacturer’s instructions. The cDNA samples were diluted ten times in RNAase-free water and stored at −20 °C until qPCR analysis.

### 2.5. Quantitative RT-PCR (qPCR)

The transcriptional levels of *LsCHS1* and *LsCHS2* were quantified by qPCR using PowerUp^TM^ SYBR Green Fast Universal PCR Master Mix (Thermo Fisher Scientific, Waltham, MA, USA). The qPCR analysis was performed using the same qPCR primers as described in a previous study using the same qPCR primers [34]. Here, the primers are listed in Table 1. The salmon louse elongation factor 1α (eEF1α), adenine nucleotide translocator 3 (ADT3), and 18S were used as the reference genes [40,41]. Reaction specificity was verified by the presence of a single peak in the melting curve. For each experiment, three to five biological replicates (three for ovaries, four for integument, intestine, oocytes, and eggs, and five for knockdown samples) were analyzed, each with two technical replicates. One sample without reverse transcriptase enzymes was included to detect possible contamination by genomic DNA. Thermal cycling and quantification were done on the Applied Biosystems 7500 Fast Real-Time PCR System in 10 μL reactions under standard conditions (initiation: 50 °C for 2 min, holding at 95 °C for 2 min, 40 cycles at 95 °C for 15 s then 60 °C for 1 min). The relative quantification analysis was performed using the 2^−ΔCt^ method by calculating the difference in threshold cycles (Ct) between the gene of interest and the average Ct values of the reference genes [42].

### 2.6. Histology

*L. salmonis* were fixed in Karnovsky fixative with 4% sucrose overnight at 4 °C. Thereafter, lice were washed twice in 1% PBS, dehydrated once in 75% ethanol, and twice in 96% ethanol for 15 min. They were then pre-infiltrated with Technovit 7100/ethanol (50/50) for two hours (Technovit 7100, Kulzer, Heraeus, Germany) and infiltrated with Technovit 7100/hardener overnight before embedding in plastic. The embedded lice were cut into 2 μm thick sections, then dried at 50 °C before they were stained with toluidine blue (1% toluidine blue in 2% borax). The slides were stained for 30 s in the staining solution, then washed in running tap water. The stained sections were mounted with DPX New Mounting Medium (Merck, Darmstadt, Germany). Photos were taken using a Zeiss Axicam 105 color (Zeiss) camera attached to a Zeiss Axio Scope.A1 (Zeiss) microscope.

### 2.7. Paraffin Embedding

Lice were paraffin embedded for in situ hybridization (see Section 2.8) and immunohistochemistry analysis (see Section 2.9). Pre-adult II and adult females were incubated in 4% paraformaldehyde diluted in phosphate-buffered saline (PBS) overnight. The next day, the lice were washed once with 1% PBS, then incubated in 1% PBS for 30 min, and subsequently kept in 70% ethanol at 4 °C for at least one day before paraffin embedding in a Histokinette (Histokinette 2000, Reichert-Jung GmbH, Nussloch, Germany). Here, the samples were washed in PBS, dehydrated through a graded ethanol series, and embedded in paraffin. The sections were cut into 5 μm thick slides using a Leica RM 225 microtome (Leica Microsystems, Wetzlar, Germany).

### 2.8. In Situ Hybridization Analysis

The in situ hybridization was performed as described earlier in [43] using a digoxigenin (DIG)-labeled kit (Roche, Basel, Switzerland) with the following modification: Proteinase K treatment was increased to 20 min. Anti-sense and sense probes of 721 bp (*LsCHS1*) and 551 bp (*LsCHS2*) were made from cDNA of *L. salmonis* with primers listed in Table 1. The sense probe was used as a negative control. The probes were visualized by using anti-digoxigenin (DIG) alkaline phosphatase fragment antigen-binding (FAB) fragment (Roche, Basel, Switzerland) and staining 1-step^TM^ NBT/BCIP plus suppressor solution (Sigma-Aldrich, St. Louis, MO, USA). Microscopy images were taken using an Axio Scope A1 light microscope connected to an Axiocam 105 (Zeiss, Oberkochen, Germany).

### 2.9. Immunohistochemistry

Chitin detection was performed by labeling paraffin-embedded *L. salmonis* with wheat germ agglutinin (WGA) lectin from *Triticum vulgaris* (Sigma-Aldrich, St. Louis, MO, USA), followed by antibody-linked staining. WGA binds specifically to *N*-acetyl-β-D-glucosamine oligomers (chitin) and *N*-acetyl-neuraminic acid (sialic acid). First, the paraffin-embedded slides (5 μm thick) were heat treated at 45 °C for 30 min. Hydration was carried out by incubating the slides first in Histo-Clear II (National Diagnostics, Atlanta, GA, USA) two times for 10 min and then in ethanol gradient (2 × 100%, 96%, 80%, 50%) for 3 min each. After the hydration steps, the slides were washed in distilled water for at least 5 min before blocking with 4% Bovine Serum Albumin (BSA) for one hour. After blocking, the slides were incubated for 45–60 min at 37 °C with 200 μL of WGA (1 μg/mL) diluted in 1% Tris Buffered Saline (TBS) buffer (Sigma-Aldrich, St. Louis, MO, USA). The negative controls were incubated in the buffer only. Thereafter, the slides were washed once in 1% TBS before 50 μL of the primary antibody (anti-WGA produced in rabbit, (Sigma-Aldrich, St. Louis, MO, USA), 1:10,000) diluted in 1% TBS-Tween (TBST) were added to each slide. The slides were incubated with the antibody for one hour at room temperature or overnight at 4 °C. After incubation with the primary antibody, the slides were washed 3× in TBS-Tween (Sigma-Aldrich, St. Louis, MO, USA) for 10 min, before the slides were incubated for 30 min in the secondary antibody diluted in TBST (goat anti-rabbit IgG, (whole molecule, Sigma-Aldrich, St. Louis, MO, USA) 1:250). After the incubation, the slides were washed 3× in TBST for 10 min, then flushed with a processing buffer (100 mM Tris-NaCl, 50 mM MgCl_2_, pH 9.5) and then incubated for 10 min in the processing buffer. After the incubation, the slides were incubated in the staining 1-step^TM^ NBT/BCIP plus suppressor solution (Thermo Scientific^TM^, Waltham, MA, USA) until the staining became visible to the naked eye (~1 min). The reaction was stopped by transferring the slides into distilled water. The slides were mounted using ImmunoHistoMount (Sigma-Aldrich, St. Louis, MO, USA), while some of the slides were rehydrated in ethanol solutions (96%, 100%, 100%) for 1 min each and washed 2x in Histo-Clear II (National Diagnostics, Atlanta, USA) for 5 min and mounted with the Histomount (Life Technologies, Carlsbad, CA, USA).

The reaction’s specificity was checked by analyzing a range of controls, leaving out the WGA, the primary, or the secondary antibody in the assay. Furthermore, some slides were treated with chitinase (*Trichoderma viride*, Sigma-Aldrich, St. Louis, MO, USA): ds*LsCHS1*-injected female lice were treated for 2 h using 2 mg/mL of chitinases, and ds*LsCHS2*-injected females for 1 h (2 mg/mL) or overnight (1 mg/mL). Control sections of adult *L. salmonis* were treated with 0.5 mg/mL chitinases for 1–3 h or overnight. The reactions were performed at 37 °C, except for overnight reactions at 4 °C. All chitinase treatments were performed in 50 mM citrate buffer pH 5.5.

### 2.10. Statistical Analysis

Statistical analysis was performed using a Student’s *t*-test or one-way analysis of variance (ANOVA), followed by Tukey’s post hoc test for multiple comparisons. Ct values obtained from the RNAi experiments were used to analyze the statistical differences between the control group and the experimental group. An independent sample *t*-test (Student’s *t*-test) (*p* < 0.05) was performed to analyze the difference in the total length of dsRNA-injected *L. salmonis* and of egg strings from dsRNA-injected female *L. salmonis* between the control and experimental groups. The differences at termination between survivors in the control and experimental groups were also calculated. One-way ANOVA and Tukey’s post hoc tests were used to analyze the significant differences between the gene expression in tissues/organs studied. Microsoft Excel 2019 was used to calculate the Student’s *t*-test. One-way analysis of variance was performed using Statistical Package for the Social Sciences (SPSS) 22.0 software (IBM, Armonk, NY, USA).

## 3. Results

### 3.1. Localization of LsCHSs

**Figure 2:**
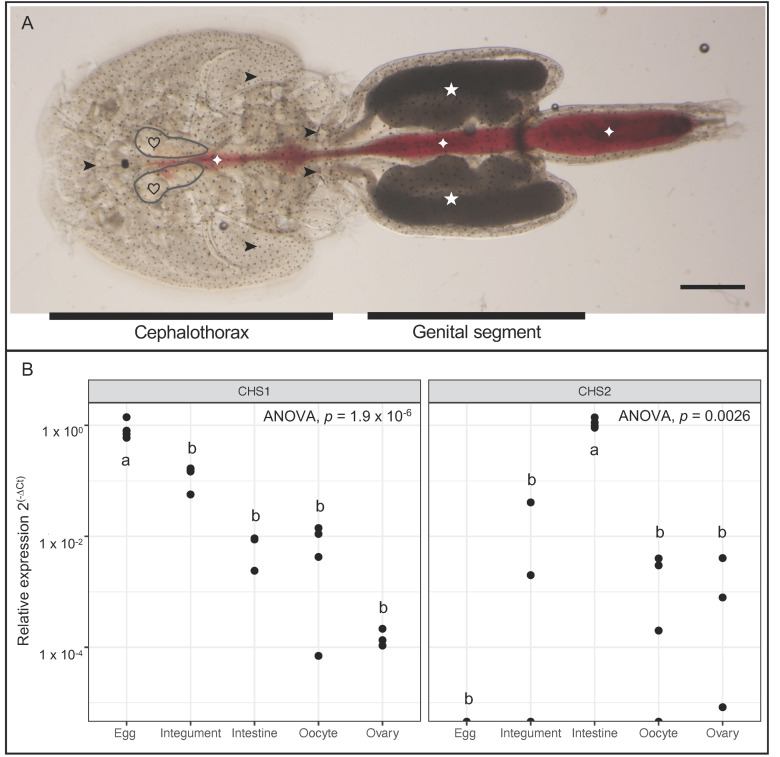
The transcriptional levels of *LsCHS1* and *LsCHS2* in tissues and organs in adult females. (**A**) Gross morphology of adult female *L. salmonis*. From the cephalothorax (ct), the ovaries (gray lines, hearts) and the integument (arrowheads) were extracted. The oocytes (stars) were extracted from the genital segment (gs). The blood-filled intestine (diamonds) extending from ct to gs was extracted. (**B**) The transcriptional levels of *LsCHS1* and *LsCHS2* were measured in dissected tissues and organs besides eggs using qPCR analysis (n = 3 biological replicates for the ovaries, and n = 4 for remaining tissues). The qPCR results were normalized to the reference genes (eEF1α, ADT3, and 18S). The relative expression levels are log-transformed, and letters indicate significant differences (*p* < 0.01, one-way ANOVA, Tukey’s test). The scale bar is 1 mm. 1 × 10^−2^.

**Figure 3:**
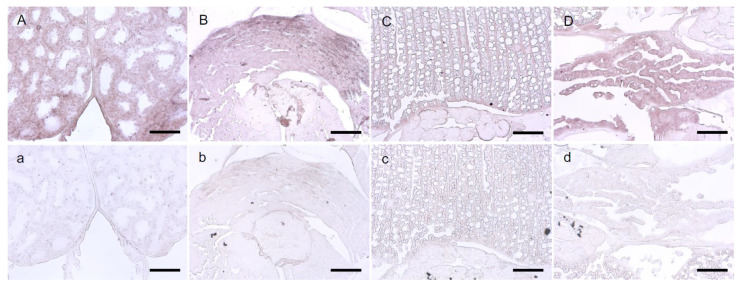
In situ hybridization of *LsCHS2* in sections of adult females. Positive stainings were detected in ovaries (**A**), muscles (**B**), intestine (**C**), and oocytes (**D**) using *LsCHS2*-specific anti-sense probes. Sense probes (**a**–**d**) show no unspecific staining. The scale bars are 100 μm.

The tissue-specific expression of *LsCHS1* and *LsCHS2* transcripts was analyzed quantitatively using qPCR and qualitatively by in situ hybridization. Expression of *LsCHS1* in adult female *L. salmonis* was found in all organs and tissue types analyzed: Integument, oocytes, ovaries, fertilized eggs, and intestine (Figure 2). *LsCHS2* was also detected in all tissues and organs tested apart from the fertilized eggs. Low levels of expression of both *LsCHS1* and *LsCHS2* was detected in ovaries and oocytes. High individual variations of *LsCHS2* levels were observed in ovaries and oocytes, likewise for *LsCHS1* in oocytes. The highest expression level was found in the fertilized eggs for *LsCHS1* and in the intestine for *LsCHS2*. In situ hybridization demonstrated *LsCHS2* in muscles, intestine, ovaries, and oocytes in adult female *L. salmonis* (Figure 3). In situ hybridization for *LsCHS1* was unsuccessful.

### 3.2. WGA Signals in Female Lice

**Figure 4:**
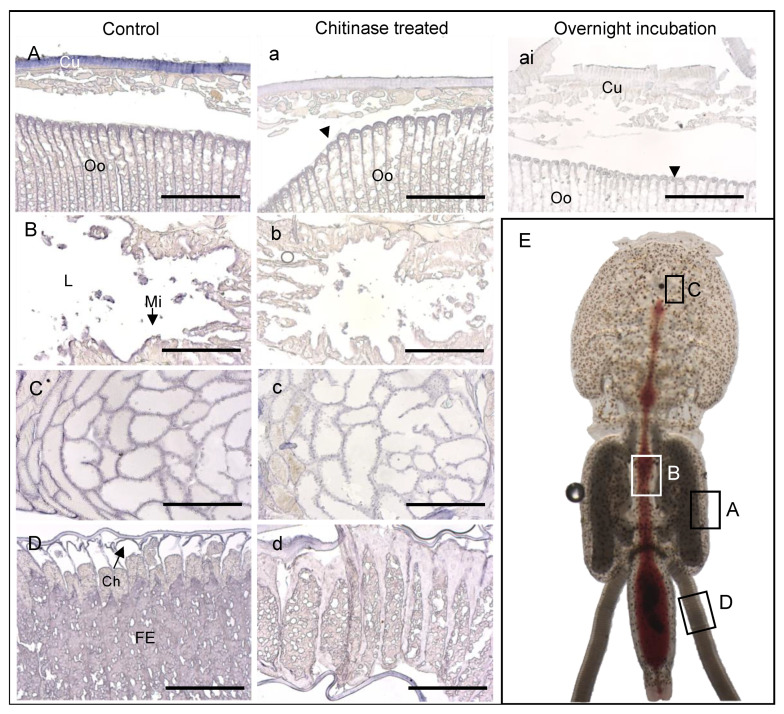
Wheat germ agglutinin (WGA) staining in female louse. WGA-labeled (paraffin) sections of control *L. salmonis* injected with ds*CPY*. (**A**) WGA stainings in oocytes of the genital segment and cuticle; (**B**) intestine, WGA staining around the microvilli (see arrow); (**C**) ovary, WGA signals around and inside the follicles; (**D**) egg string, WGA staining in the fertilized eggs, chorion (see arrow), and the layer (outermost) of the egg string. Corresponding parallel sections from the same louse treated with 0.5 mg/mL chitinases for 1 h (**a**–**c**), 3 h (**d**), and overnight (**ai**). (**E**) Gross morphology of adult female louse indicating where (**A**–**D**) are located. (a,ai) The arrowheads demonstrate the WGA signals on the edge of the oocytes. Cuticle (Cu), chorion (Ch), fertilized eggs (FE), lumen (L), microvilli (Mi), and oocytes (Oo). (**A**–**D**, **a**–**d**) The scale bars are 100 μm.

Chitin was detected in adult female *L. salmonis* using a lectin WGA assay, a commonly used method to detect chitin. The staining assay showed signals in the cuticle, ovaries, oviducts, eyes, mouth tube, papilla, around the microvilli on the midgut epithelial cells, chorion around the eggs, and egg strings (Figure 4). WGA signals were strongly reduced after chitinase treatment (Figure 4) in all tissues and organs except the ovaries (Figure 4C). Additionally, overnight digestion with chitinases was tested. After the overnight incubation with chitinases, most of the louse tissues had loosened up, and morphological structures became unrecognizable when analyzed under a microscope after mounting, especially the tissue in the cephalothorax, and could not be analyzed further. The structure of oocytes in the genital segment was, however, still intact. Overnight incubation with chitinases completely reduced the WGA signals in the cuticle and inside the oocytes (Figure 4ai). WGA signals were still found on the edge of the oocytes (Figure 4ai). A range of control stainings were performed to analyze for unspecific signals (Table S1), revealing that the primary antibody produced unspecific signals in the intestine.

### 3.3. RNAi-Mediated Knockdown of LsCHSs

**Figure 5:**
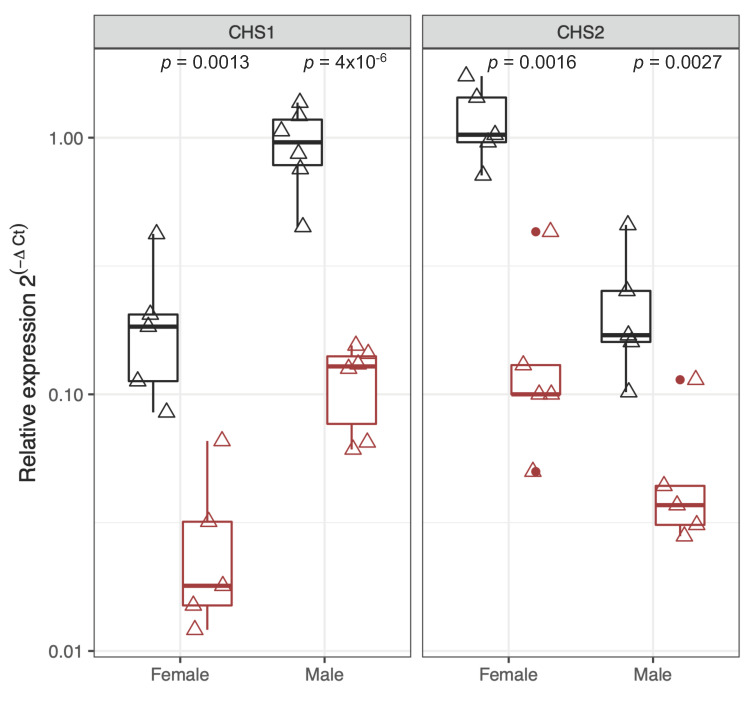
Relative expression of *LsCHS1* and *LsCHS2* in individual pre-adult II *L. salmonis* injected with ds*LsCHS1* and ds*LsCHS2* (CHS1 left and CHS2 right, brown), respectively, with comparable ds*CPY* controls (black). The qPCR results were normalized to the reference genes (eEF1α, ADT3, and 18S). One individual louse in the *LsCHS2* male group shows low knockdown effects. Each triangle represents an individual sample, and outliers are marked with a circle, (*p* < 0.01, *t*-test). The relative expression levels are log-transformed.

RNAi experiments were performed to explore the functional role of *Ls*CHS1 and *Ls*CHS2 enzymes in the parasitic stages of male and female lice. qPCR was used to confirm the silencing efficiency of dsRNA targeting either *LsCHS1* or *LsCHS2* in pre-adult II. Instar stages differ between some individual lice, causing broad gene expression variants. The analysis confirmed that *LsCHS1* was significantly (*p* < 0.01) reduced by 88% in males and 85% in females (Figure 5, left panel). Similarly, the expression of *LsCHS2* was significantly (*p* < 0.01) reduced by 71% in males and 86% in females (Figure 5, right panel).

### 3.4. Functional Impact of CHS Knockdown

Four independent RNAi trials were performed in the study: Two performed in pre-adult II females and two in pre-adult II males. In each trial, two experiments were performed, either knocking down *LsCHS1* or *LsCHS2*. The trials setups are explained in detail in Section 2.2.3.

#### 3.4.1. Knockdown of LsCHSs Induced Loss of Lice from the Fish

After dsRNA injection into the pre-adult I stage of *L. salmonis*, losses of some lice during an experiment are normal (personal observations) [41]; however, here a significant loss of lice was observed in some trials. In females (trials 3 and 4), loss of *LsCHS1* knockdown lice from the fish began as pre-adult II females reached the adult stage. Lice retrieved from filtered water outlet from the fish tanks containing *LsCHS1* knockdown lice showed morphological changes compared to controls. In trial 4, at the end of the experiment, all of the *LsCHS1* knockdown lice were lost from the host. The loss of lice after *LsCHS2* knockdown did not occur before development to the adult stage, where females with an aberrant appearance of the intestine and lack of intestinal host blood were observed in the water outlet. *LsCHS1* knockdown in males (trials 1 and 2) triggered a loss of lice, starting when they reached the adult stage. Similar morphological changes were observed in males as in *LsCHS1* knockdown females. For *LsCHS2* knockdown, no significant loss or abnormal morphology was observed in male lice.

#### 3.4.2. Knockdown of LsCHS1 Affected the Cuticle and the Subcuticular Layer

The silencing of *LsCHS1* resulted in aborted molting and abnormal cuticle formation in both female and male lice. The knockdown of *LsCHS2* did not have any observable effect on ecdysis and development in *L. salmonis.*

Development of males from the pre-adult I to the adult stage was followed to observe any effects of gene knockdown (trial 1). Two ds*LsCHS1*-injected abnormal males were found in the water outlet: One pre-adult II with incomplete molt to the adult stage, and one adult with an abnormally flexible cuticle. At termination, 56% of ds*LsCHS1*-injected males were recovered, but no adult lice, while 64% of control ds*CPY*-injected males were found, including 13% adults. In the second RNAi trial (trial 2), males injected with ds*LsCHS1* were harvested earlier to analyze if any histological changes had occurred in pre-adult II. At termination, there was no difference in survival between control and *LsCHS1*-injected lice, which both had a survival of approximately 47%. Histological analysis of the pre-adult II males revealed no abnormalities after ds*LsCHS1* injection.

**Figure 6:**
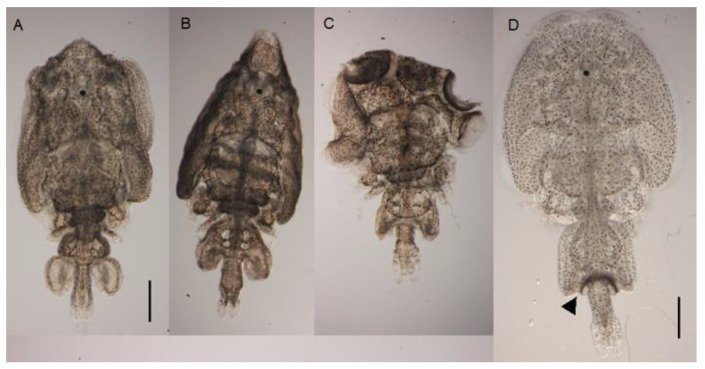
Phenotypes severity observed in female lice injected with ds*LsCHS1*. (**A**–**C**) The three phenotypes were obtained in the ds*LsCHS1* groups. (**A**) Phenotype 1, pre-adult II arrested molt to adult. (**B**) Phenotype 2, adult louse with flexible cuticle and abnormal morphology. (**C**) Phenotype 3, similar to phenotype 2 but with more dramatic changes to the morphology. (**D**) An adult female louse from the control groups with spermatophore attached to the genital segments (arrow). The scale bars are 1 mm.

RNAi knockdown of *LsCHS1* was examined in females in two separate experiments (trials 3 and 4). During trial 3, both pre-adult II and adult female lice with abnormal morphology were found in the water outlet. A total of 12 ds*LsCHS1*-injected female lice were found in the water outlet seven to eight days post-injection. These lice were unable to swim and attach to a surface. A range of phenotypes ranging from mild to more severe were observed. These were for simplicity categorized into three types: (A) Phenotype 1 (42% of the lice): Lice with incomplete molt to the adult stage. Exuvia was partially shed and typically found in the middle of the cephalothorax (Figure 6A). (B) Phenotype 2 (33 % of the lice): Lice developed to the adult stage, but with abnormally flexible cuticle and abnormal morphology of the genital segment (Figure 6B), and (C) phenotype 3 (25% of the lice): Lice similar to phenotype 2 but with more aberrant body morphology (Figure 6C).

At termination in trials 3 and 4, none of the lice injected with ds*LsCHS1* was found on the fish. For the ds*CPY*-injected control lice, 40–50 % in trials 3 and 4 recovered. Thirty-six percent of the female control lice were pre-adult II, and the rest were immature adults (T2–T3 as described by [13]).

**Figure 7:**
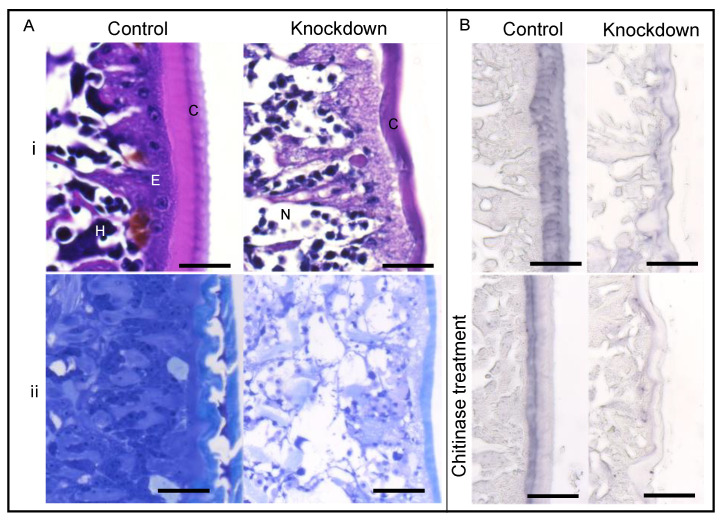
Morphology of the integument in ds*LsCHS1*-injected female lice. (**A**) Hematoxylin and erythrosin B (H&E)-stained paraffin section (panel i) and toluidine-stained plastic section (panel ii) of the cuticle and subcuticular layer in ds*CPY*- and ds*LsCHS1*-injected female lice. (**B**) WGA labeling of chitin in *CPY* or *LsCHS1* knockdown *L. salmonis* (upper) and after chitinase treatment (lower). Cuticle (C), epithelium cells (E), hemolymph (H), and necrotic tissue (N). The scale bars are 25 μm.

Histological analysis of sections revealed that the morphology of the cuticle in females injected with ds*LsCHS1* differed from control animals. Lice with phenotype 1 and 2 had two exoskeletons present in the integument. For phenotype 2, this was typically around the genital segment only, whereas in lice with phenotype 1, this could be observed both in the cephalothorax and in the genital segment. Whereas control *L. salmonis* had a single cuticle with two to three distinguishable layers, *LsCHS1* knockdown female lice had produced a thinner cuticle with no observable layers (Figure 7Ai,ii). The epithelial cell morphology in the *LsCHS1* knockdown females differed from the control lice and appeared damaged (Figure 7Ai,ii), with the cuticle detaching from the epidermis layer (Figure 7Ai). The subcuticular tissues were necrotic (Figure 7Ai,ii). The WGA staining indicated a reduced amount of chitin in the cuticle of ds*LsCHS1* compared to the control ds*CPY*-injected lice (Figure 7Bi). Before WGA staining, the *LsCHS1* and *LsCPY* knockdown female louse sections were digested with chitinases to analyze if the WGA signals in the cuticle of the *LsCHS1* knockdown females were reduced faster than the cuticle of *LsCPY* knockdown females. The chitinase treatment only reduced the WGA signals in the cuticle of the control lice, while all WGA signals were absent in the cuticle of ds*LsCHS1* lice (Figure 7Bii).

#### 3.4.3. LsCHS Knockdown Affected Blood Feeding and Growth

##### Digestion

Silencing of *LsCHS1* or *LsCHS2* induced morphological changes of the intestine and affected the feeding behavior. No abnormalities were observed in the digestion tract of males injected with ds*LsCHS1* or ds*LsCHS2* (trials 1 and 2).

The largest morphological alterations were observed in females injected with ds*LsCHS1* with phenotype 3 (Figure S1, Figure 6). Further investigation of the effect on the digestive tract was not performed. During the ds*LsCHS2*-induced knockdown in trials 3 and 4, three and four adult female lice, respectively, were collected from the water outlet with abnormal intestinal appearance. When these lice were placed on a slide under a cover-glass to study morphology further, the intestine completely disintegrated (Figure S2).

**Figure 8:**
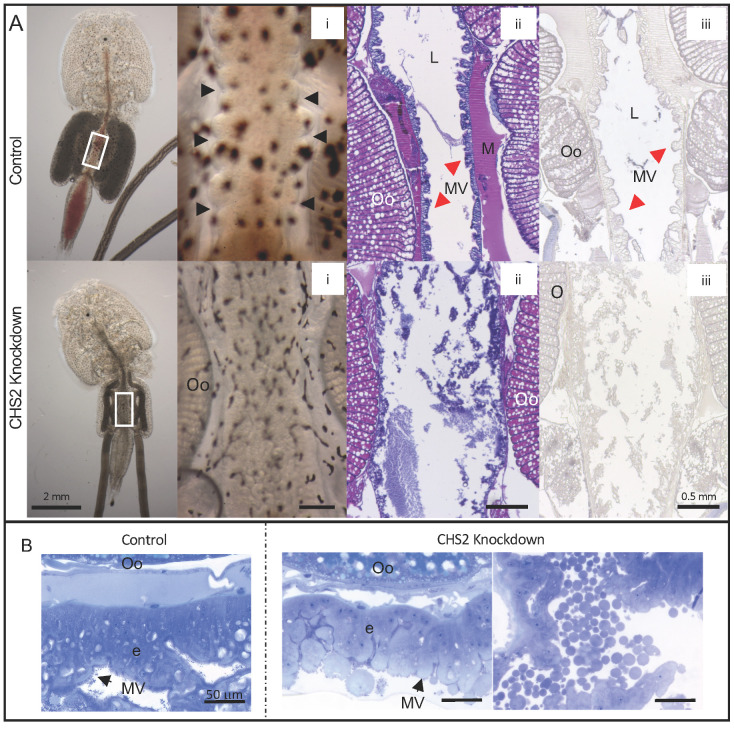
Morphology of the intestine comparing *LsCHS2* knockdown and control *Lepeophtheirus salmonis* females. Representative photos of female lice injected with either ds*CPY* (control) or ds*LsCHS2*. (**A**) The midgut of control lice (upper) and experimental lice (below). The white squares (first photo) illustrate where the other images are taken (**i**–**iii**). The intestine of the control and experimental lice (**i**); arrowheads indicate the contraction in the control intestine (**i**). H&E-stained paraffin-embedded sections (**ii**), and WGA-labeled sections (**iii**) of the midgut. The microvilli are damaged with reduced WGA signals in the intestine of the ds*LsCHS2*-injected female louse compared to the intestine of the controls. (**B**) Histopathological analysis of toluidine-stained sections of control and experimental female lice. *LsCHS2* knockdown louse epithelial cells and damaged microvilli (right image), particles were observed in the *LsCHS2* knockdown louse (left image). The lumen of the intestine (L), muscle (M), and microvilli (MV). (**A**) The scale bars are 0.5 mm (**i**–**iii**). (**B**) The scale bars are 50 μm.

At the termination of trial 3, there were no significant differences in the number of lice recovered between the control ds*CPY* and ds*LsCHS2* groups. However, in trial 4, there was a significant difference in survival between ds*LsCHS2* and control ds*CPY*-injected female lice, with three and 15 lice found on the fish, respectively (*p* < 0.05). Almost all the control lice had visible blood in the intestine, while the ds*LsCHS2* lice had no visible or a strongly reduced amount of blood in the intestine (Figure 8A). Visual analysis of live lice showed that peristaltic movements of the gut observed in control lice were completely absent in the *LsCHS2* knockdown females and the muscular contractions around the intestine (Figure 8Ai). Histological analysis revealed malformations in the midgut of ds*LsCHS2*-injected female lice ranging from a normal-looking intestine to large deformations (Figure 8Aii). In the ds*CPY* control louse sections, the muscle and microvilli were observed, while in some ds*LsCHS2* knockdown females, the amount of muscle was reduced, and microvilli were disordered. WGA staining was not observed in *LsCHS2* knockdown lice with large deformations in the intestine (Figure 8Aiii). The microvilli appeared damaged, and the epithelium cells covering the digestive tract appeared unstructured in *LsCHS2* knockdown females (Figure 8B). Histopathological alternations, including hypertrophy and desquamation, were observed (Figure 8B).

##### Growth

Both *LsCHS1* and *LsCHS2* knockdown females were significantly (*p* < 0.01) shorter than the control lice. The average length of *LsCHS1* knockdown immature adult females (~T2) was ~7 mm compared to ~9.5 mm for the control lice (T2) (Figure S3A,B). The average lengths of *LsCHS2* and *CPY* control knockdown adult lice were 10.5 mm and 12.1 mm, respectively (Figure S3A,B).

#### 3.4.4. LsCHS Knockdown Affected the Reproductive Organs and the Offspring

**Figure 9:**
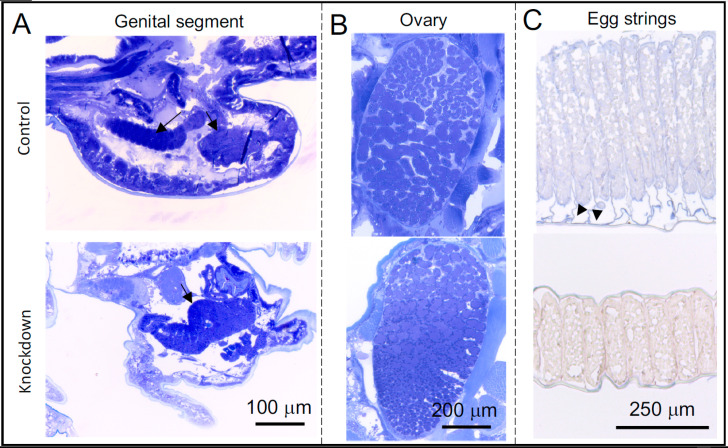
Effect of ds*LsCHS1* and ds*LsCHS2* treatment on the reproduction of female *Lepeophtheirus salmonis*. (**A,B**) Sections of *CPY* control (upper) and *LsCHS1* knockdown (below) females. (**A**) Toluidine blue-stained sections of the genital segment, the arrows indicate the oocytes. (**B**) Toluidine blue-stained sections of the ovaries. (**C**) WGA-labeled (paraffin-embedded) sections of the egg strings from *CPY* control (upper) and *LsCHS2* knockdown (below) females, the arrowheads indicate the chorion (the layer around the eggs).

The silencing of *LsCHS1* or *LsCHS2* in females (trials 3 and 4) induced changes in the ovaries, oocytes, and eggs. Females treated with ds*LsCHS1* had a malformed genital segment (Figure 6). Histological examination revealed that the *LsCHS1* knockdown females with phenotype 1 had oocytes of normal appearance, while abnormalities were apparent in the oocytes and the ovaries of lice with phenotypes 2 and 3 (Figure 9A,B). The oocyte organization in adult *LsCHS1* knockdown deviated from the control, and the follicle inside the ovaries appeared irregular and smaller. All the ds*LsCHS1*-injected lice fell off the fish before they reached the mature adult stage, and hence reproduction could not be monitored.

The oocytes of ds*LsCHS2*-injected females displayed normal morphology, but the egg strings were significantly shorter (approximately 50%) than the egg strings from the control lice (Figure S3C). The appearance of the oocytes and WGA staining signals in *LsCHS2* knockdown females did not differ from the control females (Figure S4). In female control lice, strong WGA staining was detected in the chorion, as well as in the eggs and in the outermost layer of the egg strings, indicating a chitin component there (Figure 9C). In the egg strings of *LsCHS2* knockdown females, no or faint WGA signals were observed (Figure 9C).

**Figure 10:**
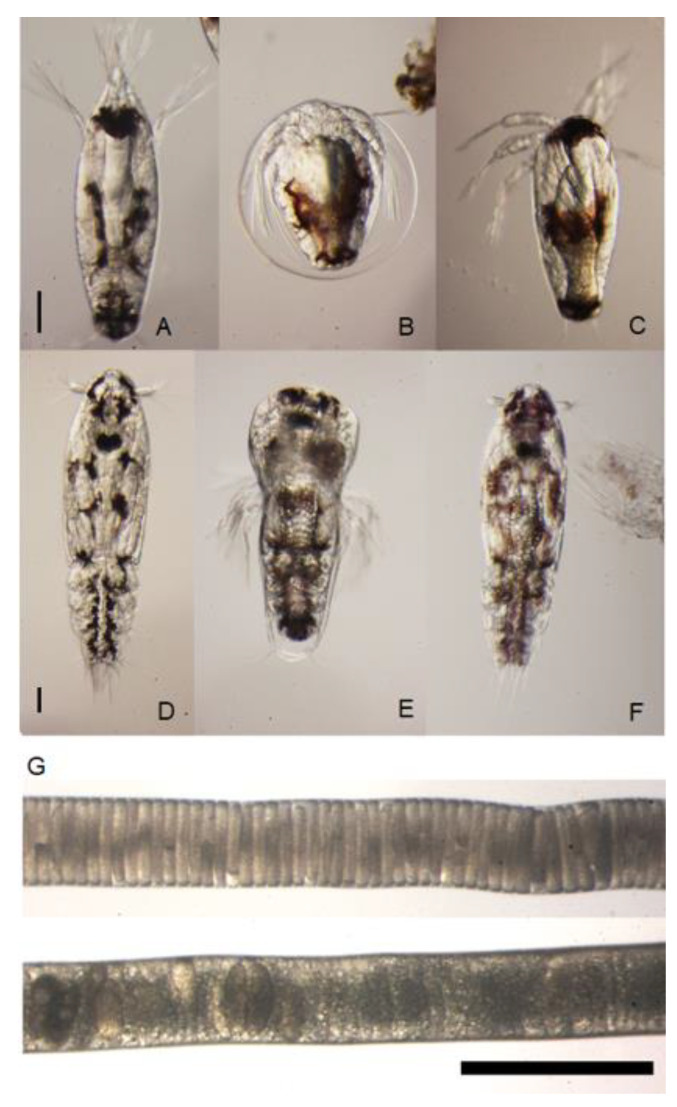
Larvae and eggs from ds*LsCHS2*-injected female *Lepeophtheirus salmonis*. Larvae from control females: Nauplius II and copepodid (**A**,**D**). Larvae from ds*LsCHS2* females from trial 3 (**B**,**C**,**E**,**F**). Larvae with incomplete hatching (**B**), nauplius II arrested molt completely (**C**), copepodid arrested molt during ecdysis (**E**), and copepodid with an abnormal intestine (**F**). The second set of egg string pairs from adult female ds*CPY* (above) and ds*LsCHS2* (below) lice from trial 4 (**G**). (**A**–**F**) The scale bars are 0.1 mm and (**G**) 1 mm.

At the termination of trial 3, most females carried eggs: Six of nine lice injected with ds*LsCHS2*, and 11 of 12 control lice. Larvae from ds*LsCHS2*-treated females, however, exhibited reduced hatching success (Figure 10B), arrested molt to nauplius II (Figure 10C), or developed into copepodids with partially shed exuvia (Figure 10E) or with air bubbles inside the intestine (Figure 10F). All control larvae had normal development (Figure 10D). In trial 4, three egg strings were collected from the ds*LsCHS2* females, but none hatched. Larvae from the control group developed normally. The structure of one of the egg strings produced by an *LsCHS2* knockdown female from trial 4 is shown in Figure 10G.

Reproduction in females on fish together with ds*LsCHS1*-injected males was not followed. Females placed on fish together with ds*LsCHS2*-injected males reproduced normally and had viable offspring.

## 4. Discussion

*Lepeophtheirus salmonis* possesses two CHSs, *Ls*CHS1 and *Ls*CHS2. RNAi-mediated knockdowns of *CHSs* have shown that CHS1 and CHS2 are required for development, survival, egg hatching, oviposition, and oogenesis in diverse insects [29,36,37,44,45], which is similar to phenotypes obtained in insects treated with benzoylurea [46,47].

### 4.1. Chitin Detection by WGA and In Situ Localization of LsCHS2

We detected chitin in the cuticle, eyes, ovaries, oviduct, oocytes, papilla, mouth tube, epithelial cells in the gut, and the fertilized egg string of *L. salmonis.* Chitin components have also been reported in these tissues and organs in pancrustaceans [48,49,50,51].

WGA staining is commonly used to analyze chitin levels. WGA staining has been documented in the integument and midgut of several arthropods [49,50,52,53,54], in the egg, eggshell, and ovaries of *Aedes aegypti* [48], and the reproductive organs in the copepods *Oithona nana* and *Oithona similis* [49]. Similar to our results in adult females, WGA staining has been detected in the cuticle, intestine, and reproductive organs of the copepods *Oithona nana* and *Oithona similis*. WGA staining was also detected in the reproductive system of *Oithona* males [49].

In situ hybridization of *CHS*s has been performed in a few insect species. Here, *CHS2* was located in situ in the midgut epithelial cells, similar to what has been shown for insect CHS2 [16,55,56]. However, *CHS2* localization in muscles or reproductive organs has not been reported in insects, like we could observe here, indicating that *Ls*CHS2 has additional functions compared with insects.

### 4.2. LsCHS1 and LsCHS2 are Also Expressed in the Reproductive Organs and Intestine

From the first studies on CHSs in insects, it was assumed that CHS1 was specific to the integument and CHS2 to the midgut; however, later studies have shown that both CHSs are expressed in the reproductive system. Similarly to our findings, CHSs have been reported in the eggs of the mosquitoes *A. gambiae* [56] and *Culex pipiens pallen* [57] and in the ovaries and eggs of the planthopper *Sogatella furcifera* [58], as well as in the ovaries of the triatomine bug *Rhodnius prolixus* [20] and oriental armyworm *Mythimna separata* [58]*. LsCHS1* and *LsCHS2* were not highly expressed in the ovaries and oocytes but their importance for reproduction in *L. salmonis* possibly earlier in development cannot be excluded. The expression of *LsCHS1* in fertilized eggs can indicate the importance for embryogenesis in *L. salmonis*. Similar to insect CHS2, the expression of *LsCHS2* was not detected in fertilized eggs [59].

We could detect *LsCHS1* mainly in the integument, while *LsCHS2* was mainly found in the intestine of adult female *L. salmonis*. These results are comparable to insect *CHS1* and *CHS2*, e.g., in the beetle *Leptinotarsa decemlineata* [60], the moth *Manduca sexta* [28], the beet armyworm *Spodoptera exigua* [61,62], and the mosquito *Anopheles gambiae* [56]. *CHS1* and *CHS2* expression in insects has also been reported in the intestine and integument, respectively. In the intestine of insects, *CHS1* is mainly found in the foregut and hindgut, not midgut [56,60], and *CHS2* has been reported to be expressed at low levels in the integument of *Ostrinia furnacalis* [63] and *M. sexta* [28]. Similar to what we observed in the present study, *LsCHS1* was expressed at low levels in the intestine and *LsCHS2* in the integument. The result presented in this study shows that *LsCHS1* and *LsCHS2* are mainly expressed in the integument and intestine, respectively. Nevertheless, both can also be found in the same tissues and organs, namely, in the integument, intestine, and reproductive organs. However, concluding from our RNAi-mediated knockdown, *LsCHS1* and *LsCHS2* have different functions. A previously published study also supports this finding, which includes knockdown experiments of these genes in *L. salmonis* larvae [35].

### 4.3. Silencing the Expression of LsCHS1 Disrupts Development and Growth

In insects, it is well documented that CHS1 is important for molting and development, while CHS2 for feeding and possibly egg production. In *L. salmonis*, silencing the expression of *LsCHS1* resulted in abnormal molting and the reduction of chitin in the exoskeleton, while the silencing of *LsCHS2* did not affect ecdysis or exoskeleton chitin formation. The silencing of *LsCHS1* did not affect the molting of pre-adult I lice, while most pre-adult II lice exhibited abnormal molting to the adult stage. It is unlikely that the two molt processes are significantly different, but the protein level of Ls*CHS1* may still have been sufficient in pre-adult I to accomplish molting. Similar results are reported for *LsCHS1* knockdown *L. salmonis* larvae, where only the second molt after treatment was affected [35]. Suppressing the expression of *LsCHS1* induced changes in the cuticle structure. Some of the pre-adult lice were partially trapped inside the exuviae. In contrast, others molted to malformed adults with an incomplete exoskeleton and some with remains of the old exoskeleton. RNAi-mediated knockdown of insect CHS1 showed similar phenotypes with incomplete molt, e.g., the planthopper *S. furcifera* [58], the fruit fly *Bactrocera dorsalis* [64], the beetle *L. decemlineata* [60], and the pea aphid *Acyrthosiphon pisum* [44]. The WGA assay also supports the role of *Ls*CHS1 in the synthesis of chitin in the exoskeleton, similar to insect CHS1.

### 4.4. Silencing the Expression of LsCHS1 or LsCHS2 Interferes with the Digestive System

RNAi knockdown of both *LsCHS1* and *LsCHS2* in female lice induced changes in the intestine of the experimental lice. The *LsCHS1* knockdown adult females exhibited abnormal morphology of the intestine. In insects, CHS1 synthesizes chitin into the hind- and foregut. If *Ls*CHS1 also has a similar role, this could explain the large morphological changes observed in the intestine of adult lice [56,60]. The silencing of *LsCHS2* interfered with blood feeding, and the epithelial cells covering the intestine in adult female *L. salmonis* were damaged. Interestingly, no such effect was found in males, which could indicate that females are more dependent on a high expression of *LsCHS2*, possibly due to a higher digestive requirement for energy consumption required in the production of eggs. In some mosquitoes, blood feeding is required for egg production, and male mosquitoes do not feed on blood [65]. Yet, it is presently unknown whether blood feeding is necessary for reproduction in females. Blood can be observed in male *L. salmonis*, but it is currently unknown whether they are obligate blood feeders. *LsCHS2* is highly expressed in pre-adult I and adult females; however, in males, the expression is several folds lower (Figure 5), which could explain why only the females had an abnormal phenotype. In trial 3, the intestine in the females was less damaged than the intestine in females in trial 4, suggesting that the damage occurs with time. The females in trial 4 were approximately two weeks older than females in trial 3.

Silencing the expression of *CHS2* in insects affects the synthesis of the peritrophic matrix and damages the gut epithelium; the insects stop feeding and eventually die [29,37]. Both effects are in accordance with the putative functional similarity between *L. salmonis* and insects. However, *L. salmonis* do not seem to have a peritrophic matrix; nonetheless, they express intestine-specific *CHS* [18,66]. Insects that lack the peritrophic matrix are reported to have only one copy of *CHS* [20,44,67]. Many of these insects belong to the order Hemiptera and have instead a perimicrovillar membrane, an extracellular layer covering the intestinal microvilli. Unlike the peritrophic matrix, the perimicrovillar membrane increases the absorption capacity of nutrients from diluted diets [19]. As demonstrated here in *L. salmonis*, WGA staining in the intestinal microvilli may not be conclusive, as our results show unspecific staining in the intestine. However, after chitinase treatment, the WGA signals were reduced in the lice intestine, and the signals were mostly absent in the intestine of the ds*LsCHS2* knockdown females. Taken together, these results support the role of *LsCHS2* in synthesizing chitin in the midgut. In the triatomine bug *R. prolixus*, which lacks the peritrophic matrix, chitin is present around the midgut epithelium [50]. Based on the expression of *LsCHS2* and our *LsCHS2* knockdown results, it is possible that *L. salmonis* has a layer around the microvilli with similar protection and function as the peritrophic matrix or perimicrovillar membrane. Still, more investigation is needed before this can be shown conclusively.

The intestine’s muscular contractions were absent in *LsCHS2* knockdown females, and our histological investigation showed that the muscles around the intestine appeared smaller than in control animals. However, further investigation is also needed here before clear conclusions can be drawn.

### 4.5. LsCHS1 and LsCHS2 Are Important for Reproduction

Silencing *CHS* expression in insects by RNAi induces changes in reproductive organs and functions such as oogenesis, oviposition, egg morphology, and hatching [20,21,36,45]. In *L. salmonis*, silencing the expression of either of the two *CHS*s affected the reproductive system but in different ways. Knocking down *LsCHS1* caused structural alterations in the ovaries and oocytes, while *LsCHS2* knockdown interfered with the hatching of offspring and changed the morphology of extruded eggs. Reproductive effects could not be demonstrated in *LsCHS1* knockdown females, as they never reached the mature egg-producing female stage. However, the malformation in the reproductive organs indicates that these lice could not produce viable offspring. Insect CHS1 is shown to be required for embryonic development [45,68]. It could be speculated that *Ls*CHS1 is important for the embryonic cuticle formation as the expression of *LsCHS1* is high in non-treated maturing eggs.

Silencing the expression of *LsCHS2* in females showed that the majority of the larvae in the first pair of egg strings hatched (trial 3), while the larvae from the second pair of egg strings (trial 4) did not hatch. The difference in the duration of each trial, or differences in the level of knockdown, between the females could explain the differences in reproduction success. In insects, *CHS2* knockdown in the beetle *T. castaneum* and the boll weevil *Anthonomus grandis* also reduced reproduction [36,37], which was caused by starvation of the mother due to a dysfunctional peritrophic matrix. The females in trial 3 had most likely enough energy to produce some offspring. The few females that survived in trial 4 were apparently not eating, and normal egg production was inhibited. A reduction of chitin in the chorion in the egg strings of *LsCHS2* knockdown lice could also explain the unsuccessful hatching. Similar results are reported for *dsCHSA*-treated *T. castaneum*, where the amount of chitin was dramatically reduced in the eggs, and hatching did not take place [36]. In *R. prolixus,* the eggs produced by *dsCHS*-treated females had different morphology, and a reduction of chitin content was found in the previtellogenic and vitellogenic oocytes [20]. We could see a reduction of chitin in the ovaries in *LsCHS1* knockdown females, but not in *LsCHS2* knockdown females. The oocytes in the ds*LsCHS2* knockdown and the control lice seem to have a similar amount of chitin.

The offspring from *LsCHS2* knockdown lice may have undergone molting arrest during larval development due to a lack of nutrients caused by impaired feeding in the mother. Blood is a nutrient-rich food supply that may be optimal for egg production and development of the offspring. Lacking essential components during the development of larvae could influence developmental success. In conclusion, the roles of chitin synthases in parasitic copepod *L. salmonis* resemble the described roles in insects. The two CHSs appear to play diverse roles, with *Ls*CHS1 mainly being involved in exoskeleton construction and *Ls*CHS2 in intestinal function. Limited knowledge on CHSs is available from copepods, and future studies should investigate the role of these enzymes in molting, reproduction, and intestine.

## Figures and Tables

**Table 1:** List of primer sequences used for quantitative RT-PCR (qPCR) assay, in situ hybridization, and RNA interference (RNAi) approach.

**Gene**	**Primer Identification**	**Forward (3′–5′)**	**Reverse (3′–5′)**	**Method**	**Product Size**
*LsCHS1*	Forward_b2874	GCGTTGCGTTCATACCTTCT	TAATTTTCCCACCAACCCGC	qPCR	214
	Reverse_b2875				
	Forward_b4615	TAATACGACTCACTATAGGGAGA-	CGGTGCCAAACGTTCACAAT	In situ	721
	Reverse_b4614	AGCCTGGACCGTACCTGTAT		anit-sense probe	
	Forward_b4613	AGCCTGGACCGTACCTGTAT	TAATACGACTCACTATAGGGAGA-	In situ	721
	Reverse_b4616		CGGTGCCAAACGTTCACAAT	sense probe	
	Forward_b4611	TAATACGACTCACTATAGGGAGA-	TAATACGACTCACTATAGGGAGA-	dsRNA	380
	Reverse_b4612	TGGTGTGAGGCGTTAGAACC	CGTGAGTGGAGTGGCTTCAT		
**Gene**	**Primer Identification**	**Forward (3′–5′)**	**Reverse (3′–5′)**	**Method**	**Product Size**
*LsCHS2*	Forward b2876	TCACTCACGTCCCCATTTCT	TCGATGGATGCTAGCCGAAT	qPCR	242
	Reverse b2877				
	Forward_b7044	CTTGGACACTTCCTTTAGGC	TAATACGACTCACTATAGGGAGA-	In situ	551
	Reverse_b5763		GACCGCTGCATAAGATACG	anti-sense probe	
	Forward_b5762	TAATACGACTCACTATAGGGAGA-	GACCGCTGCATAAGATACG	In situ	551
	Reverse_b7045	CTTGGACACTTCCTTTAGGC		sense probe	
	Forward b2843	TAATACGACTCACTATAGGGAGA-	TAATACGACTCACTATAGGGAGA-	dsRNA	563
	Reverse b2844	CAACGAACCCACGAAGAGTTGATT	TTGTCGTCCCGTTAATATAGGCCA		

## Data Availability

Data is contained within the article or supplementary material.

## Supplementary Materials

Supplementary materials can be found at https://www.mdpi.com/2075-1729/11/1/47/s1. Table S1: Overview of the results from the WGA assays. Each line represents one assay. Plus (+) indicates the reagents used in a single analysis, and minus (−) negative controls. In the signal column, plus indicates staining, red reduced staining, and minus no staining, and antibody (Ab), Figure S1: Effects on ovaries in *LsCHS1* knockdown females. (**A**) H&E-stained and (**B**) WGA-labeled sections of control and *LsCHS1* knockdown females, Figure S2: *LsCHS2* knockdown female with completely disintegrated intestine: (**A**) Overview photo of the louse carrying eggs; (**B**) photo taken of the louse without coverslip; and (**C**) with cover slip. (**A**) Scale bar 1 mm (6.25x magnification), and (**B**,**C**) 12.5x magnification, Figure S3: The total length: (**A**) *LsCHS1* knockdown females; (**B**) *LsCHS2* knockdown females; and (**C**) egg strings from *LsCHS2* knockdown females compared with comparable controls, Figure S4: Effects on genital segment in *LsCHS2* knockdown females; (**A**) H&E-stained sections; (**B**) toluidine blue-stained sections; and (**C**) WGA-labeled sections of control and *LsCHS2* knockdown female lice.
