# Supplementary material for: Chitin Synthases Are Critical for Reproduction, Molting, and Digestion in the Salmon Louse (Lepeophtheirus salmonis)
Source: Life (Basel). 2021 Jan 13;11(1):47. doi: 10.3390/life11010047 (PMC7828418; doi:10.3390/life11010047)
Supplement: Supplementary file 1 [file life-11-00047-s001.zip › life-1017801-supplementary-for XML/Supplementary files/Table S1.pdf]

| Tissue | Treatment | | | | Signals |
|---|---|---|---|---|---|
| | Chitinase | WGA | 1°Ab | 2°Ab | |
| | + | + | + | + | Red |
| | - | + | + | + | + |
| Cuticle | - | - | + | + | - |
| | - | + | - | + | - |
| | - | + | + | - | - |
| | + | + | + | + | Red |
| | - | + | + | + | + |
| Oocytes | - | - | + | + | - |
| | - | + | - | + | - |
| | - | + | + | - | - |
| | + | + | + | + | Red |
| | - | + | + | + | + |
| Intestine | - | - | + | + | + |
| | - | + | - | + | . |
| | - | + | + | - | + |
| | + | + | + | + | Red |
| | - | + | + | + | + |
| Ovaries | - | - | + | + | - |
| | - | + | - | + | - |
| | - | + | + | - | - |
| | + | + | + | + | Red |
| | - | + | + | + | + |
| Egg string | - | - | + | + | - |
| | - | + | - | + | - |
| | - | + | + | - | - |

| Tissue | | Treatment | | |
|---|---|---|---|---|
| | Chitinase | WGA | 1°Ab | 2°Ab |
| | + | + | + | + |
| | - | + | + | + |
| Cuticle | - | - | + | + |
| | - | + | - | + |
| | - | + | + | - |
| | + | + | + | + |
| | - | + | + | + |
| Oocytes | - | - | + | + |
| | - | + | - | + |
| | - | + | + | - |
| | + | + | + | + |
| | - | + | + | + |
| Intestine | - | - | + | + |
| | - | + | - | + |
| | - | + | + | - |
| | + | + | + | + |
| | - | + | + | + |
| Ovaries | - | - | + | + |
| | - | + | - | + |
| | - | + | + | - |
| | + | + | + | + |
| | - | + | + | + |
| Egg string | - | - | + | + |
| | - | + | - | + |
| | - | + | + | - |

| Signals |
| --- |
| |
| Red |
| + |
| - |
| - |
| - |
| Red |
| + |
| - |
| - |
| - |
| Red |
| + |
| + |
| - |
| + |
| Red |
| + |
| - |
| - |
| - |
| Red |
| + |
| - |
| - |
| - |
