# Supplementary figures and images for: Chitin Synthases Are Critical for Reproduction, Molting, and Digestion in the Salmon Louse (Lepeophtheirus salmonis)
Source: Life (Basel). 2021 Jan 13;11(1):47. doi: 10.3390/life11010047 (PMC7828418; doi:10.3390/life11010047)

**A****Control****CHS1 knockdown****H&E-staining**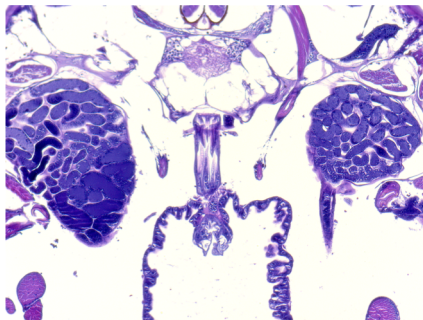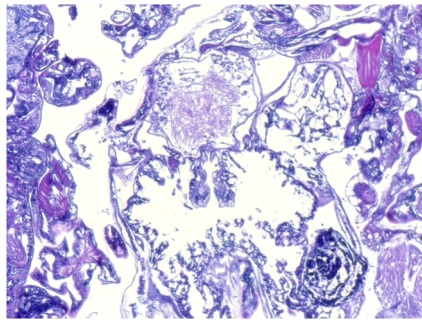**WGA-staining**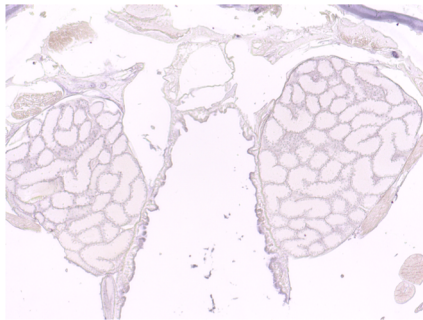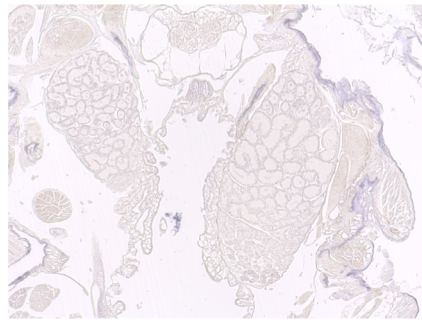

Supplement: Supplementary file 1 [file life-11-00047-s001.zip › life-1017801-supplementary-for XML/Supplementary files/Figure S1.pdf]

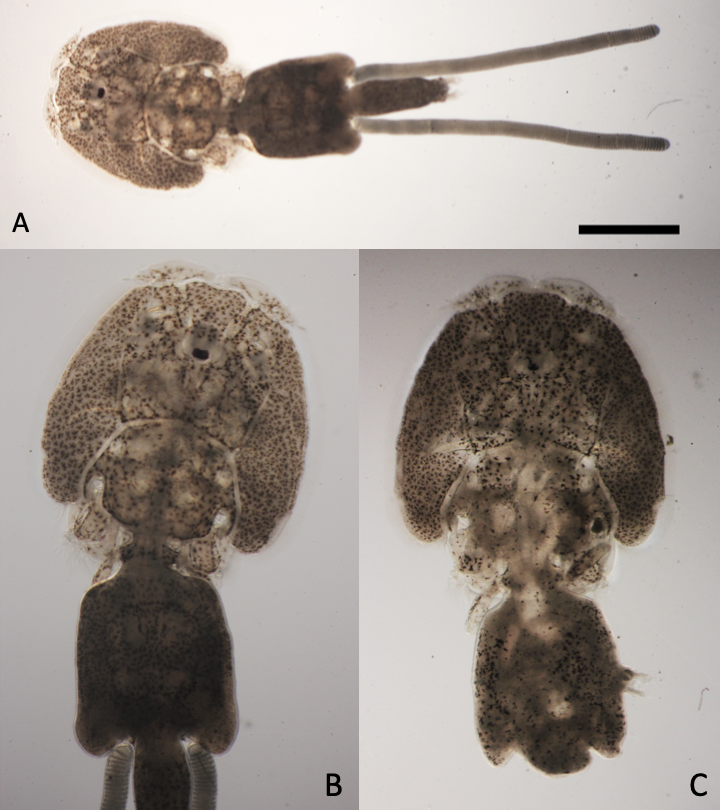

Supplement: Supplementary file 1 [file life-11-00047-s001.zip › life-1017801-supplementary-for XML/Supplementary files/Figure S2.tiff]

**A**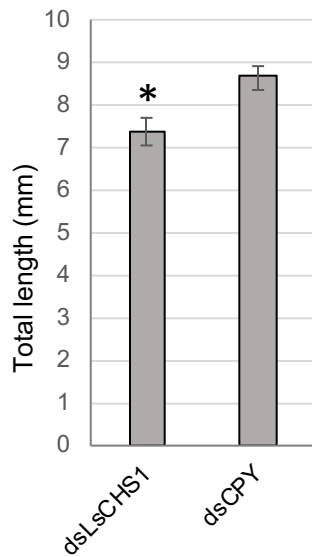**B**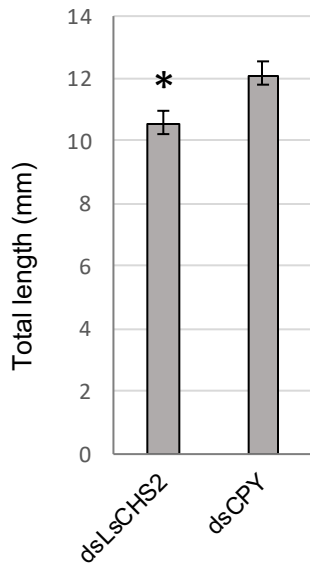**C**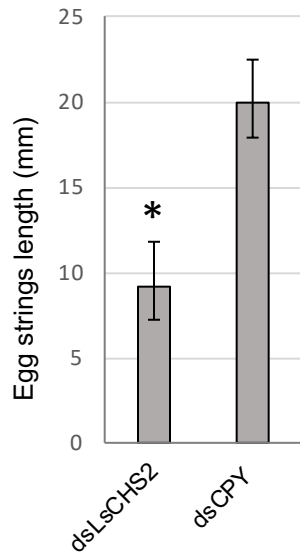

Supplement: Supplementary file 1 [file life-11-00047-s001.zip › life-1017801-supplementary-for XML/Supplementary files/Figure S3.pdf]

A

Control

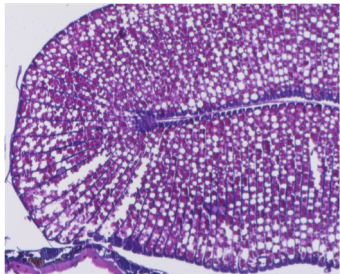

B

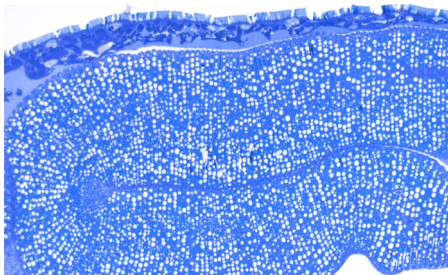

C

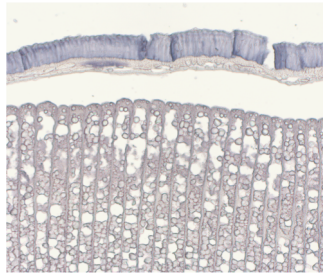

CHS2 knockdown

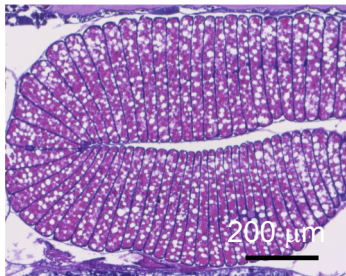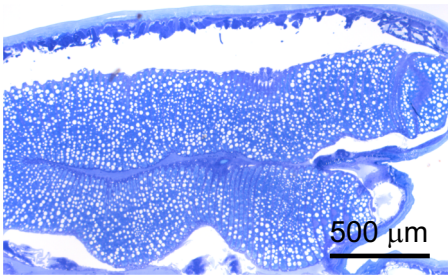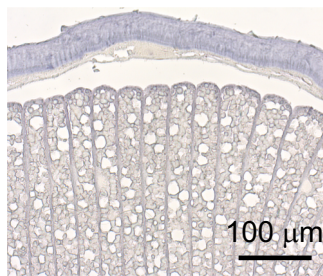

Supplement: Supplementary file 1 [file life-11-00047-s001.zip › life-1017801-supplementary-for XML/Supplementary files/Figure S4.pdf]
